# Supplementary material for: Soy Protein Isolate–Chitosan Nanoparticle-Stabilized Pickering Emulsions: Stability and In Vitro Digestion for DHA
Source: Mar Drugs. 2023 Oct 22;21(10):546. doi: 10.3390/md21100546 (PMC10608249; doi:10.3390/md21100546)
Supplement: Supplementary file 1 [file marinedrugs-21-00546-s001.zip › marinedrugs-2622648-supplementary.pdf]

Supplementary Materials

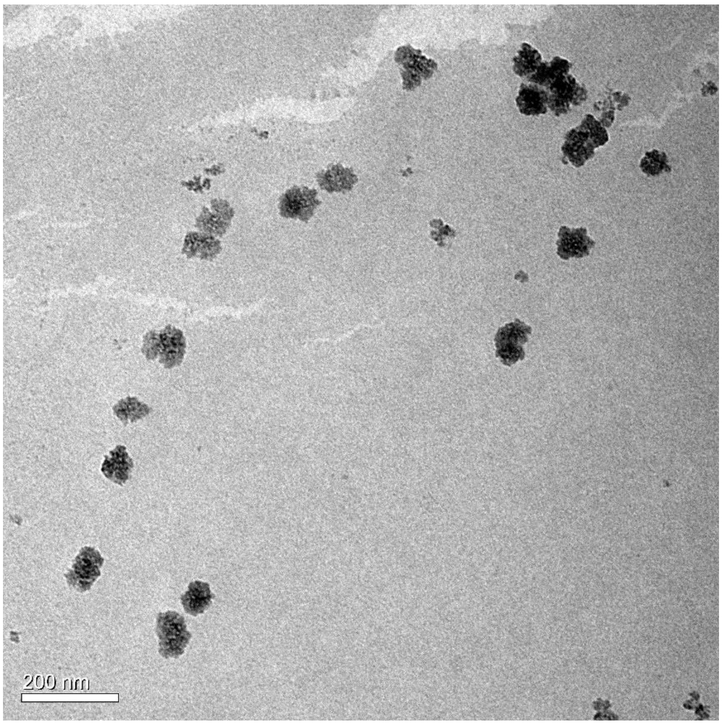

Figure S1. TEM image of SPI-CS nanoparticles. (In a chitosan solution concentration of 0.5 mg/mL at pH 5.4, a soybean protein isolate solution concentration of 2% at pH 7.0, a volume ratio of 5:8 between the two solutions, and a chitosan molecular weight of 5 kDa.)

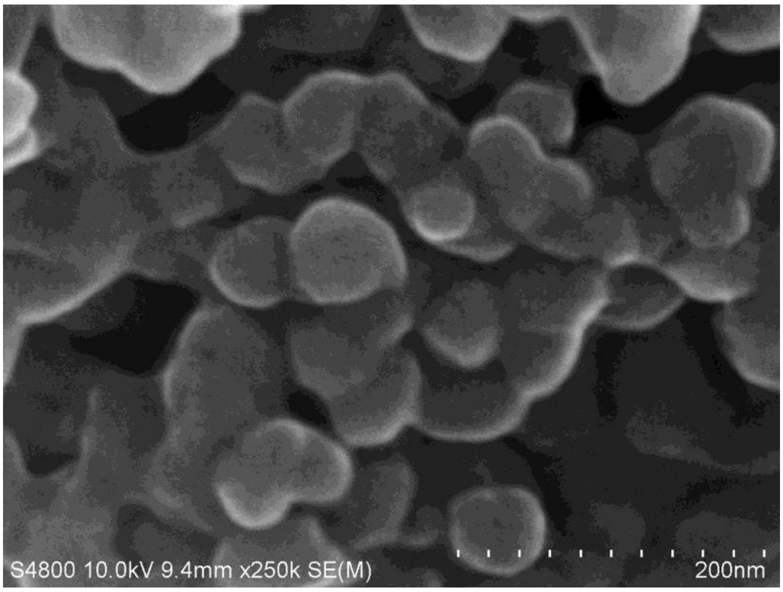

Figure S2. SEM image of SPI-CS nanoparticles. (In a chitosan solution concentration of 0.5 mg/mL at pH 5.4, a soybean protein isolate solution concentration of 2% at pH 7.0, a volume ratio of 5:8 between the two solutions, and a chitosan molecular weight of 5 kDa.)

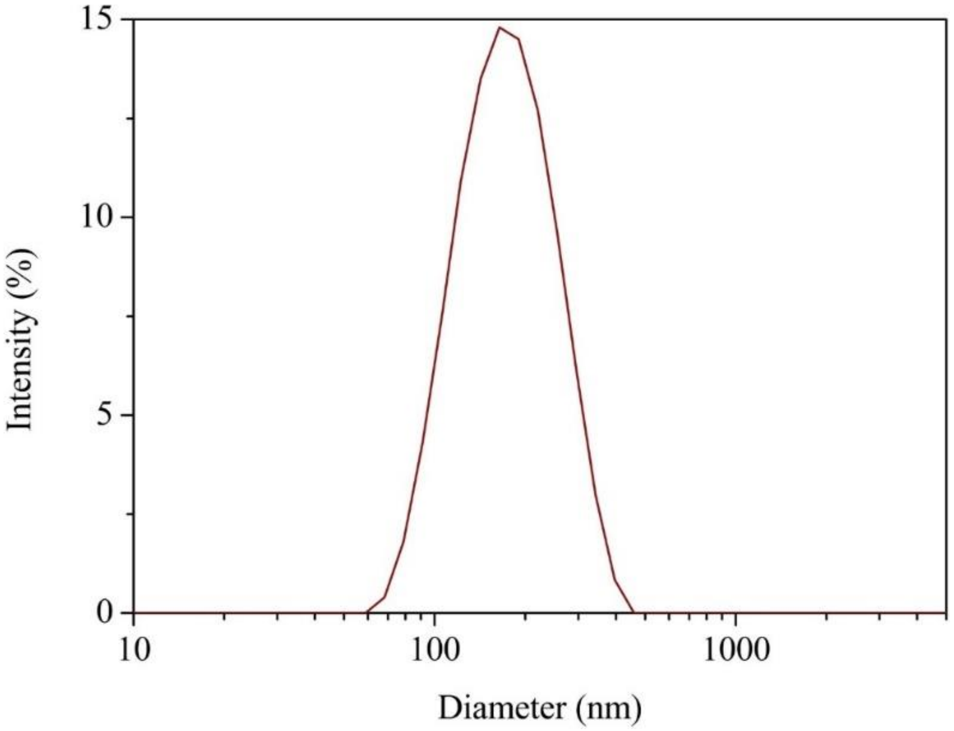

Figure S3. The particle size distribution of SPI-CS nanoparticles. (In a chitosan solution concentration of 0.5 mg/mL at pH 5.4, a soybean protein isolate solution concentration of 2% at pH 7.0, a volume ratio of 5:8 between the two solutions, and a chitosan molecular weight of 5 kDa.)
